# How to detect propaganda from social media? Exploitation of semantic and fine-tuned language models

**DOI:** 10.7717/peerj-cs.1248

**Published:** 2023-02-20

**Authors:** Muhammad Shahid Iqbal Malik, Tahir Imran, Jamjoom Mona Mamdouh

**Affiliations:** 1 Department of Computer Science, School of Data Analysis and Artificial Intelligence, Higher School of Economics, Moscow, Russia; 2 Department of Computer Science, Capital University of Science and Technology, Islamabad, Pakistan; 3 Department of Computer Sciences, College of Computer and Information Sciences, Princess Nourah bint Abdulrahman University, Riyadh, Saudi Arabia

**Keywords:** Propaganda, News articles, word2vec, BERT, Binary model, Semantic, Linguistic, LSA

## Abstract

Online propaganda is a mechanism to influence the opinions of social media users. It is a growing menace to public health, democratic institutions, and public society. The present study proposes a propaganda detection framework as a binary classification model based on a news repository. Several feature models are explored to develop a robust model such as part-of-speech, LIWC, word uni-gram, Embeddings from Language Models (ELMo), FastText, word2vec, latent semantic analysis (LSA), and char tri-gram feature models. Moreover, fine-tuning of the BERT is also performed. Three oversampling methods are investigated to handle the imbalance status of the Qprop dataset. SMOTE Edited Nearest Neighbors (ENN) presented the best results. The fine-tuning of BERT revealed that the BERT-320 sequence length is the best model. As a standalone model, the char tri-gram presented superior performance as compared to other features. The robust performance is observed against the combination of char tri-gram + BERT and char tri-gram + word2vec and they outperformed the two state-of-the-art baselines. In contrast to prior approaches, the addition of feature selection further improves the performance and achieved more than 97.60% recall, f1-score, and AUC on the dev and test part of the dataset. The findings of the present study can be used to organize news articles for various public news websites.

## Introduction

Democracies and social destruction are greatly influenced by propaganda, which permeates every aspect of our daily lives. It is difficult to determine whether an article is biased or neutral in this era because there are so many news platforms that appear to present neutral and biased articles. Authors may not be intentionally biased about any topic, or they may do it on purpose to capture readers’ thinking about a specific topic. Propaganda appears in different fields such as psychology, sociology, history, and political science due to its diverse nature in different domains. Each discipline defines ’propaganda’ differently.

Propaganda can detract our minds when we completely believe in the information presented in an article. Likewise, it is extremely difficult for a person to determine whether the article’s contents are propagandistic or not (Baeza-Yates, 2018). We have a prominent example of propaganda, which had been used in the 2016 US Presidential elections (Mueller, 2018). The study by Rashkin et al. (2017a) introduced the word n-gram technique for propaganda detection, but the author(s) concluded that n-gram did not classify accurately when out-of-domain articles are used. Likewise, Barrón-Cedeño et al. (2019) used word and char n-gram features and concluded that a hybrid combination of these features has a significant impact on propaganda detection. Recently, Barfar (2022) used linguistic features with coalitional game theory and achieved 84% f1-score. However, to the best of our knowledge, few attempts are done to address the imbalance issue of the datasets but the results are not significant. Likewise, comparisons of various textual, semantic, word embeddings, language, and topic models features are rarely conducted. Additionally, class balancing along with feature selection techniques can drastically improve classification performance.

To handle these challenges, we built a binary classification model for the identification of propaganda and non-propaganda news articles. Nine types of features are explored to investigate their impact on propaganda detection. The features are part-of-speech, word uni-gram, char tri-gram, linguistic, LSA, word2vec, fine-tuning of BERT, FastText, and ELMo models. A publicly available Qprop dataset (Barrón-Cedeño et al., 2019) is used for the experimental setup. Decision tree, naïve Bayes, and random forest are compared to describe the best classifier for the given task. As the dataset is imbalanced, three state-of-the-art oversampling methods are investigated to define the best method. In addition, filter and wrapper-based feature selection techniques are employed to further increase the classification accuracy. Theoretically, the findings of this research add valuable contributions to prior literature by investigating the impact of the latest language model, word embeddings, semantics, and linguistic features for propaganda identification. In addition, it motivates researchers to explore new contextual and textual characteristics to build a more robust propaganda detection model.

The main highlights of this study are presented below:

• The latest state-of-the-art word embedding, topic, and language models (Word2vec, FastText, fine-tuning of BERT, ELMo, and LSA) are explored to develop a robust detection model.
• The fine-tuning of BERT revealed that BERT-320 sequence length in the presence of SMOTE ENN demonstrated promising metrics.
• The comparison of machine learning (ML) techniques revealed that random forest demonstrated the best performance.
• Experiments revealed that the char tri-gram as a standalone model and char tri-gram + BERT as a hybrid combination demonstrated the best performance and outperformed the two baselines.
• SMOTE ENN oversampling model demonstrated significant improvement when applied to the latest but imbalanced Qprop English news articles dataset.
• Our proposed framework in the presence of SMOTE ENN and wrapper feature selection methods achieved 97.78% recall, 97.93% f1-score, and 97.93% AUC.

The remaining part of the article is organized as follows: Section 2 presents the review of related studies on propaganda identification. Section 3 describes the methodology adopted by the proposed framework. The results are presented in section 4, and related discussion and implications are discussed in Section 5. Finally, Section 6 provides the conclusion and directions for future work.

## Related Work

From the perspective of feature extraction, most of the prior studies used stylistic and linguistic representations, which are topic or genre-independent. Different features have different roles, however, the content length and topic features have a very little role, but among significant features, char n-gram is one of them (Stamatatos, 2009). Prior studies have explored such types of characteristics for the identification of bias and propaganda and these types of features are more influential and significant. The study by Popat et al. (2017) utilized stylistic features to verify the credibility of claims from the news articles. The features were extracted using manually-designed lexicons. In comparison, our research mainly focuses on some textual, word embeddings and language models. Most probably, stylometric features are useful to identify hyperpartisanship. The study in Potthast et al. (2017) investigated the stylometric models to distinguish between real/fake news articles. They developed a dataset containing real-time news articles taken from nine different sources. The stylometric technique was first developed by Koppel, Schler & Bonchek-Dokow (2007) to predict factuality and bias for the verification of authorship. They hypothesized that biased contents have a typical writing style. Another case study (Horne & Adali, 2017) developed a model to identify fake news. Their conclusion revealed that the use of proper nouns and title structure are very important characteristics in contrasting fake from real news. Likewise, another method (Horne & Adali, 2017) was presented to distinguish between real, fake, and satire news by using writing style and complexity indicators. The authors concluded that ‘fake news’ has low readability indices, shorter lengths, uses simple language, and less technical words.

Rashkin et al. (2017b) designed a corpus of news articles extracted from English Gigaword and seven different news sources. They concluded that word n-grams dropped the performance when tested on unseen sources. Later, a binary propaganda classification model was developed by Shao et al. (2017) and validated on a publicly available dataset of 15k bots. Their model achieved a 95% AUC with random forest. Likewise, Horne, Khedr & Adali (2018) developed an identification model using 130 content features collected from the literature. In 2019, a multi-granularity neural network-based analysis is conducted on a big dataset (Da San Martino et al., 2019). They focused on sentence-level and fragment-level classification. Their method achieved 60.82% and 22.58% f1-scores on sentence-level and fragment-level classification. Then, suspicious terms from radical content are identified by Nouh, Nurse & Goldsmith (2019) using the ML methods. Their outcome revealed that random forest is the best classifier yielding an accuracy of 94%. Later, a binary propaganda identification model is designed (Barrón-Cedeño et al., 2019). They compiled a Qprop dataset which consists of real news articles gathered from 104 different news outlets. Their evaluation revealed that char n-gram produced the best results when combined with Nela. Likewise, other approaches such as Oliinyk et al. (2020) and Altiti, Abdullah & Obiedat (2020) used TF-IDF, POS, lexicons-based, and word vectors for propaganda identification.

Recently, a model is designed to identify propagation-based fake news (Han, Karunasekera & Leckie, 2020) using the graph neural network method. They evaluated their model on both seen and unseen datasets. In addition, it is also explored that fake-news propagation from news media is the main root of the generation of inter-religion conflicts (Baugut & Neumann, 2020). More recently, a two-step system is introduced to identify the propaganda in news articles (Li et al., 2021). Experiments on the 550 news articles exhibited that their model outperformed the state-of-the-art methods. Similarly, another effort is done using TF-IDF and bag of words features (Khanday, Khan & Rabani, 2021) and achieved an f1-score of 0.58% for propaganda text. Then, Polonijo, Šuman & Šimac (2021) proposed a method to combine sentiment scores with the word2vec method and claimed that this combination of semantic and emotional content results in improving the propaganda identification task. The interpretable propaganda detection method was presented by Yu et al. (2021). The authors demonstrated that their interpretable features could be combined with pre-trained language models to improve accuracy. We selected their study as the second baseline for comparison. Other approaches include a spinning language model (Bagdasaryan & Shmatikov, 2021), mixed-code text with deep learning (Tundis, Mukherjee & Mühlhäuser, 2021), topic-modeling with fuzzy logic (Mukhamediev, Filatova & Yakunin, 2021), visual and textual content with a balanced dataset (Guo & Vosoughi, 2021), linguistic features (Barfar, 2022) and fake news (Huang et al., 2022). The summary of related studies is presented in Table S1.

By summarizing the literature, we find the following gap in the literature:

• Lack of significant feature engineering: The majority of the prior works used either textual characteristics or contextual characteristics for propaganda identification. A lack of comparison between both types of approaches is found.
• Lack of feature selection: Few studies used filter-based feature selection techniques to improve the propaganda detection performance but their findings are inconsistent and contradictory.
• Lack of class balancing approaches: According to our knowledge, very few studies used basic class balancing methods.

In the article, we contribute in these directions by using significant linguistic, word embeddings, semantic, and language-context approaches and comparing their performance with existing approaches; by using filter and wrapper-based feature selection methods to further improve the performance; and by using class balancing approaches to balance the news articles dataset and to further improve the classification performance.

## Framework Methodology

**Figure 1:**
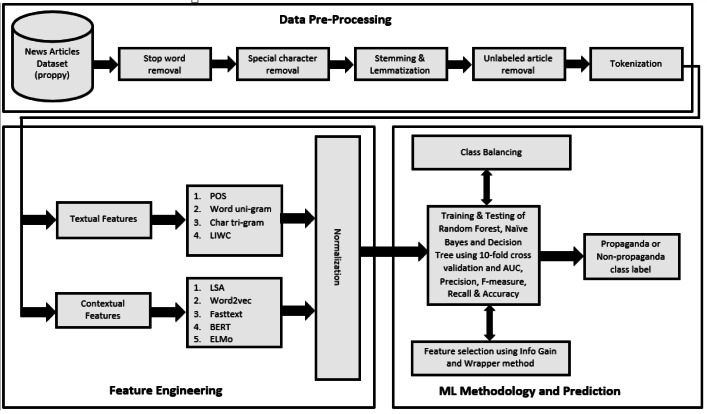
Pipeline for propaganda detection model for news articles.

In this section, the pipeline of the proposed framework is presented and detail of its component is provided. In addition, the description of the dataset, proposed features, applied ML methods, and evaluation metrics are presented. The overall methodology is presented in Fig. 1. The Qprop dataset (news articles dataset, publicly available) is used and pre-processing steps are applied on it. After that, nine types of features are extracted using their standard methods and min-max normalization is employed to scale the features within a certain range. Then experiments are conducted using naive Bayes, decision tree, and random forest ML methods, 10-fold cross-validation, and four evaluation metrics to train and evaluate the binary classification model. The binary model is then considered for the class balancing approach to balance the dataset. Furthermore, the feature selection process is applied to further improve the identification performance. The components of the pipeline shall be discussed in detail in the subsections.

### Dataset description

For the experimental setup, we use a publicly available Qprop dataset (Barrón-Cedeño et al., 2019), that consists of real-life published news articles by different news outlets. The Qprop has three parts. *i.e.,* Train, Dev, and Test. The train part has 4,021 propaganda, and 31,972 non-propaganda instances. Likewise, Dev has 575, and 4,560, and Test has 1,141, and 9,021 propaganda and non-propaganda instances. The reason why we selected all three parts is that baseline 1 (Barrón-Cedeño et al., 2019) used all of them for experimentation. In this way, the performance of our proposed framework can be easily compared with the baseline.

### Machine learning models and evaluation metrics

For the binary classification task, we choose the Python language for the development of ML models and training and testing purposes. The naïve Bayes, decision tree, and random forest methods are chosen for the experimental setup. These are the popular ML methods and are demonstrated comparatively better in other domains. The classification results will be evaluated using five evaluation metrics, *i.e.,* accuracy, precision, recall, f1-measure, and Area under Curve (AUC). All sort of experiments is conducted using the 10-fold cross-validation method.

### Proposed features

We employed nine types of features to investigate their impact on propaganda identification in news articles. The detail of each type of feature is given below.

#### Part of speech

Within the structure of a particular language, part of speech (POS) is the categorization of words based on similar grammatical properties, roles, and functions. The most common part of speech is “noun, verb, adjective, adverb, pronoun, preposition, conjunction, interjection, numeral, and article”. However, the Stanford NLP toolkit (Manning et al., 2014) provides 36 tags for part of speech. We used this toolkit and generated 36 features.

#### Word N-gram

They are also called lexical bundles or multi-word expressions, a set of co-occurring words within a given window. N-gram is usually a sequence of N words in a given sample of text (Brown et al., 1992). This model always predicts the occurrence of a word based on the occurrence of N-1 prior words, often used in natural language processing and text mining tasks. We only extracted uni-gram and bi-gram from the corpus.

#### Char N-gram

This model is used to embed textual sequence, that is based upon uni, bi, or tri-gram characters. This model can predict the occurrence of the next character as well as the sequence in a given sentence. We only used char tri-gram in our feature set and dropped uni and bi-grams because uni and bigrams did not demonstrate better performance (Cavnar & Trenkle, 1994).

#### Linguistic inquiry and word count

In 2007, Chung & Pennebaker (2012) proposed a robust framework for the analysis of verbal and written text samples, called Linguistic Inquiry and Word Count (LIWC). It is a lexicon-based feature extraction tool and its master dictionary is composed of approximately 6,400 words. More than 100 features are extracted using this library.

#### Latent semantic analysis

For analyzing semantic relationships between the set of documents (news articles, product reviews, etc.) and the terms they contain, the LSA technique supports this facility in NLP (Landauer, Foltz & Laham, 1998). The outcome shall be a set of topics/concepts. A matrix is constructed that consists of word counts per topic/document in which rows represent unique words and columns represent each topic/document. We used a 100-topic/document threshold in training the model.

#### Word2Vec

Word embeddings are capable of capturing the context of a word in a document and it is one of the most popular representations of text vocabulary. Word embeddings are vector representations of a particular word. Among other methods, word2vec is one of the most popular methods to learn and generate word embeddings (Goldberg & Levy, 2014). Word2vec supports two models, *i.e.,* continuous bag of words (CBOW) and skip-gram. We used the CBOW model and 100 dimensions for the training of the word2vec model.

#### Fine-tunning of BERT

Jacobin Devlin and his colleagues designed a language representation model named Bidirectional Encoder Representations from Transformers (BERT) at Google lab in 2018 (Devlin et al., 2018). This model presented a very promising performance on classification and regression tasks. In addition, it enables us to do faster development on fewer data processing requirements with improved results (Devlin et al., 2018). We are interested to fine-tune the BERT model for propaganda identification. There are a few steps involved in fine-tuning and we adopted the following steps: To input the data for the BERT model, it must be transformed into a specific format. For example, the text of the news articles is first tokenized by using the uncased-BERT tokenizer. After creating the word tokens, a special token is appended at the start and a special token is added at the end, that is [CLS]. Before classification, the tokens are mapped to their indexes. After that sequence length is configured. We selected 64, 128, 256, 384, and 512 (six sequence lengths for fine-tuning) sequence lengths for the experimental setup, and each news article is truncated/padded to a single fixed length. Finally, we developed attention masks to distinguish between padded and real tokens.

Regarding the fine-tuning of the BERT model for the classification of propaganda articles, all relevant parameters (attention mask, class labels, and tokens) are aggregated into the Tensor matrix. The hyperparameters are given in Table 1, which was used to fine-tune the base model of BERT.

#### ELMO

ELMo is an open-source deep contextualized word representation (Peters et al., 2018) that could be trained on a large text corpus. Previous embedding models generate spelling-oriented embeddings but ELMo generates context-sensitive embeddings. It generates numerous embeddings for a single word that enhance the probability of a word that can be used in multiple places according to the context. We have implemented ELMo using the TensorFlow library with 5 epochs and a learning rate of 0.1.

#### FastText

It is a method that was developed by the Facebook research team for learning word embeddings and text classification. It works like a word2vec embedding model but word2vec generates embedding for a single word based upon its spellings whereas fast text generates embeddings based upon the sum of character n-gram vector. Once a word is represented in character n-gram then embeddings could be attained using a skip-gram model (Bojanowski et al., 2017). We used a pre-trained FastText model to investigate the impact of generated dimensions on propaganda identification.

### Baseline

For comparison with state-of-the-art baselines, we selected two studies from prior literature: Barrón-Cedeño et al. (2019) and Kausar, Tahir & Mehmood (2020). We choose only outperforming feature combinations from both baselines to compare with our framework. The detail of outperforming combination from each baseline is given below:

**Table 1:** Hyperparameters for fine-tuning BERT.

**Sequence length**	**Batch size**	**Epochs**	**Learning rate**	**Epsilon (eps)**
64	32	4	2e−5	1e−8
128	32			
256	32			
320	32			
384	16			
512	8

1. Char n-gram + Nela from Barrón-Cedeño et al. (2019).
2. Char n-gram + Nela + Word n-gram from Kausar, Tahir & Mehmood (2020).

## Experiments and Evaluation

In this section, we conducted three sets of experiments to investigate the impact of the proposed framework and the comparison between features and baselines for propaganda identification in news articles.

### Experiment 1: Fine-tunning BERT with and without class balancing

The section describes two types of experiments, conducted to fine-tune the BERT model to come up with the best performance along with the optimum configuration of the parameters for binary classification in the news articles. The first experiment performs the fine-tuning of the BERT model without class balancing and the second experiment performs the fine-tuning with class balancing techniques.

Tables 2 and 3 show the performance of various models of BERT for binary classification obtained by fine-tuning using the given hyperparameters (Table 1). The metrics chosen to evaluate the performance are accuracy, precision, recall, and f1-score. We executed the fine-tuning of BERT four times to investigate the impact of class balancing on BERT and binary classification (micro-average). The first experiment is without class balancing, the second is with random oversampling, the third is with the SMOTE (Synthetic Minority Over-sampling Technique) method (Chawla et al., 2002) and the fourth experiment is with the SMOTE ENN method (Guan et al., 2021). The reason why we applied class balancing techniques is that the Qprop dataset is highly imbalanced, therefore we want to investigate the impact of three oversampling methods on binary classification. The first one is random oversampling which is the naïve method. It creates new samples of minority class by duplicating the actual class’s instances and it does not add new samples at all. The second method is SMOTE introduced by Chawla et al. (2002) and it is an over-sampling technique. It works by using the KNN ML model to create synthetic data. The starting point is to choose random instances from the minority class, after that k of the nearest neighbors for randomly selected instances are configured. This procedure repeats until both classes have equal instances. In contrast, SMOTE ENN method combines the SMOTE with the ENN technique. One of the limitations of SMOTE is that it can generate boundary and noisy instances and can lead to overfitting. The ENN method can serve as a cleaner/filter method to remove instances of those classes whose labels differ from the labels of at least two of its three nearest neighbors. Thus, SMOTE ENN method can reduce the possibility of overfitting.

**Table 2:** Performance comparison (micro-average) of fine-tuned BERT classifiers with random oversampling, SMOTE, and SMOTE ENN methods on the dev part of the dataset.

**Class Balancing**	**Features**	**Precision**	**Recall**	**F1-Score**	**AUC**
Without balancing	BERT-64	90.27	79.09	84.58	79.14
		BERT-128	92.92	81.88	86.94	82.07
		BERT-256	92.72	81.48	86.75	81.98
		BERT-320	94.67	84.28	88.64	84.97
		BERT-384	92.35	81.03	86.59	81.25
	BERT-512	93.79	82.58	87.81	83.08
With random oversampling	BERT-64	90.29	83.34	86.68	86.04
		BERT-128	92.94	86.13	89.41	88.97
		BERT-256	92.74	85.73	89.1	88.88
		BERT-320	94.69	88.53	91.51	91.87
		BERT-384	92.37	85.28	88.68	88.15
	BERT-512	93.81	86.83	90.19	89.98
With SMOTE oversampling	BERT-64	90.39	86.48	88.39	88.09
		BERT-128	93.04	89.27	91.12	91.02
		BERT-256	92.84	88.87	90.81	90.93
		BERT-320	94.79	91.67	93.2	93.92
		BERT-384	92.47	88.42	90.4	90.2
	BERT-512	93.91	89.97	91.9	92.03
With SMOTE ENN oversampling	BERT-64	90.48	88.88	89.67	88.27
		BERT-128	93.13	91.67	92.39	91.2
		BERT-256	92.93	91.27	92.09	91.11
		BERT-320	94.88	94.07	94.47	94.1
		BERT-384	92.56	90.82	91.68	90.38
	BERT-512	94.00	92.37	93.18	92.21

**Table 3:** Performance comparison (micro-average) of fine-tuned BERT classifiers with random oversampling, SMOTE, and SMOTE ENN methods on the test part of the dataset.

**Class balancing**	**Features**	**Precision**	**Recall**	**F1-Score**	**AUC**
Without Balancing	BERT-64	90.87	79.4	84.78	79.17
		BERT-128	93.30	81.71	87.12	82.33
		BERT-256	92.91	81.58	86.80	82.24
		BERT-320	94.85	84.46	88.82	85.18
		BERT-384	92.80	81.23	86.60	81.67
	BERT-512	94.20	82.65	87.83	83.25
With random oversampling	BERT-64	90.88	83.73	87.16	85.97
		BERT-128	93.31	86.04	89.53	89.13
		BERT-256	92.92	85.91	89.28	89.04
		BERT-320	94.86	88.79	91.72	91.98
		BERT-384	92.81	85.56	89.04	88.47
	BERT-512	94.21	86.98	90.45	90.05
With SMOTE oversampling	BERT-64	90.93	86.67	88.75	88.04
		BERT-128	93.36	88.98	91.12	91.2
		BERT-256	92.97	88.85	90.86	91.11
		BERT-320	94.91	91.73	93.29	94.05
		BERT-384	92.86	88.5	90.63	90.54
	BERT-512	94.26	89.92	92.04	92.12
With SMOTE ENN oversampling	BERT-64	91.12	89.06	90.08	88.24
		BERT-128	93.55	91.37	92.45	91.4
		BERT-256	93.16	91.24	92.19	91.31
		BERT-320	95.1	94.12	94.61	94.25
		BERT-384	93.05	90.89	91.96	90.74
	BERT-512	94.45	92.31	93.37	92.32

Although we generated four tables describing training loss, validation loss, validation accuracy, and other parameters for each time to fine-tune the BERT model, we added only those results that are more relevant. Therefore, the best results are picked against each sequence length, and the results of five BERT classifiers (each with a different sequence length) on both the dev and test parts of the Qprop dataset are shown in Tables 2 and 3. On the dev part of the dataset, BERT classifiers with large sequence lengths performed better than those with short sequence lengths. For example, BERT with the shortest sequence length of 64 gives the worst precision, recall, f1 score, and AUC (90.27, 79.09, 84.58, 79.14) respectively. In contrast, all BERT classifiers with 128, and higher sequence lengths presented much better performance. The best performance is observed from BERT with a sequence length of 320 against all four metrics. In addition, the same findings can be seen on the test part of the Qprop dataset. Moreover, sequence length is the main parameter that enables the BERT classifier to achieve promising results.

Likewise, random oversampling, SMOTE, and SMOTE ENN methods were applied one by one on both parts of the Qprop dataset and fine-tunings of BERTs are again carried out three times. The results are demonstrated in Tables 2 and 3. The performances of BERT classifiers with class balancing on the dev part are presented in Table 2. It is evident from the results that with SMOTE ENN oversampling, the BERT-320 sequence length showed the very highest metric values (at least 94%). This validates the outperformance of SMOTE ENN oversampling method as compared to SMOTE and random oversampling. The worst performance is observed with the random oversampling method because it only duplicates the minority class samples and does not add variety. Likewise, the performance on the Test part of the Qprop dataset is demonstrated in Table 3. The BERT classifiers with sequence lengths of 64, 128, 256, 320, 384, and 512 are fine-tuned considering three oversampling methods. Again, the BERT classifier with 320 sequence length gives the highest metric values with SMOTE ENN oversampling method. Moreover, the metric values are higher than the metric values obtained on the dev part of the Qprop dataset. Thus, SMOTE ENN oversampling is outperforming when we did fine-tuning of the BERT model, in contrast, random oversampling showed the worst performance.

### Experiment 2: Classification performance and comparison of features

In this section, several experiments are conducted to compare the performance of three ML models to identify only propaganda class, using the dev and test parts of the Qprop dataset (Barrón-Cedeño et al., 2019). Ten-fold cross-validation and five evaluation metrics (accuracy, recall, precision, f1-measure, and AUC) are used for the experimental setup. In addition, naïve Bayes, decision Tree, and random forest ML models are selected to define the best-performing model.

In the first experiment, we compared the performance of three ML models on eight types of features (part-of-speech, LIWC, word uni-gram, char tri-gram, word2vec, ELMO, Fast text, and LSA feature models). Here, only propaganda class is considered and we are interested to analyze the performance of ML models in identifying propaganda class. The Dev and Test parts of the dataset are used for validation and the training part is used for training the ML models. The performance of each classifier in accuracy, precision, and f1-score metrics on eight features is presented in Table 4. By analyzing thoroughly, it is clearly shown that the random forest model demonstrated the best performance in accuracy, precision, and f1-score metricses on all features validated on the dev and test parts of the dataset. The reason behind outperformance of random forest is its strengths: (1) it is an ensemble model, trained with the bagging methodology, (2) can learn non-linear decision boundary properly and handles outliers by binning them. Moreover, it demonstrated best performance for classfication and regression tasks in other domains (Mehboob & Malik, 2021). The decision tree model demonstrated itself as the second-best model in identifying propaganda class and naïve Bayes gives the worst performance. For the next experiments, we will use the random forest model.

**Table 4:** Comparison of three ML classifiers for identifying only propaganda class.

Metric	**Features**	**Dev**			**Test**		
		Naïve Bayes	Decision tree	Random forest	Naïve Bayes	Decision tree	Random forest
Accuracy	Part-of-Speech	81.48	85.45	88.9	81.59	85.69	89.03
		LIWC	79.2	87.36	90.60	79.33	87.49	90.93
		Word Uni gram	63.73	88.59	91.60	63.83	88.68	91.95
		ELMO	79.54	90.81	92.89	79.77	90.96	92.94
		FastText	80.49	91.78	93.30	80.68	91.82	93.57
		Latent Semantic Analysis	81.52	92.69	94.21	81.76	92.82	94.42
		Word2vec	83.87	93.78	95.60	83.97	93.89	95.82
	Char Tri Gram	84.89	95.49	96.40	84.93	95.68	96.62
Precision	Part-of-Speech	27.35	32.2	50.0	28.65	33.76	50.34
		LIWC	24.26	42.12	79.2	25.78	43.65	79.58
		Word Uni gram	30.02	47.35	86.7	31.65	48.67	86.95
		ELMO	38.24	50.21	88.56	39.29	51.45	88.94
		FastText	42.70	50.89	90.12	43.87	51.93	91.32
		Latent Semantic Analysis	50.16	53.32	91.4	51.34	54.67	91.75
		Word2vec	56.07	57.79	92.9	57.19	58.89	93.26
	Char Tri Gram	66.87	78.85	94.1	67.29	79.59	94.45
F1-Score	Part-of-Speech	32.04	29.23	33.75	33.08	30.32	33.93
		LIWC	31.1	39.31	41.54	31.97	39.87	41.88
		Word Uni gram	35.15	43.23	45.04	35.39	43.49	45.51
		ELMO	37.2	47.3	49.3	37.43	47.58	49.59
		FastText	38.78	47.61	49.2	38.94	47.85	49.48
		Latent Semantic Analysis	40.12	49.04	51.28	40.33	49.29	51.46
		Word2vec	45.89	50.1	52.83	45.97	50.34	52.94
	Char Tri Gram	56.9	78.32	81.60	57.12	78.52	81.78

The second experiment is set up with 10-fold cross-validation, random forest (as it performed best in prior experiments), and four evaluation metrics (precision, recall, f1-score, and AUC). Only propaganda class is considered a class label. The objective is twofold: (1) To investigate the impact of three class balancing methods on nine types of features for propaganda class as a standalone model, and (2) to investigate the impact of various combinations of proposed features in the presence of SMOTE ENN oversampling and comparison with baselines for propaganda class identification. By addressing the first objective, the results are demonstrated in Tables 5 and 6, respectively. Table 5 presents the performances on the dev part and Table 6 presents the performances on the test part of the dataset. It is apparent from Tables 5 and 6 that the char tri-gram gives the best performance in all four evaluation metrics on the dev as well as test parts of the dataset as a standalone model. The comparison of nine types of features including BERT-320 is shown in the upper parts of both Tables 5 and 6. However, BERT-320 has comparable performance with char tri-gram in precision metrics, but not in other evaluation metrics.

**Table 5:** Impact of class balancing on proposed features for identifying only propaganda class on the dev part of the dataset.

**Class balancing**	**Features**	**Precision**	**Recall**	**F1-Score**	**AUC**
Without	Part-of-Speech	50.0	7.0	33.75	53.0
		LIWC	79.2	21.2	41.54	60.2
		Word Uni gram	86.7	29.6	45.04	64.4
		ELMO	88.56	29.7	49.3	64.9
		FastText	90.12	29.9	49.2	65.14
		Latent Semantic Analysis	91.4	30.8	51.28	65.3
		Word2vec	92.9	34.1	52.83	66.8
		BERT-320 (fine-tuned)	93.27	44.45	59.28	75.38
	Char Tri Gram	94.1	72.0	81.60	84.2
With random oversampling	Part-of-Speech	70.54	64.34	67.3	72.54
		LIWC	77.29	77.68	77.48	77.78
		Word Uni gram	86.85	81.47	84.07	81.76
		ELMO	88.79	82.65	85.61	82.87
		FastText	90.37	85.79	88.02	86.89
		Latent Semantic Analysis	91.7	86.67	89.11	87.67
		Word2vec	93.1	87.07	89.98	88.56
		BERT-320 (fine-tuned)	93.76	87.67	90.61	89.21
	Char Tri Gram	94.19	87.82	90.89	90.39
With SMOTE	Part-of-Speech	72.78	66.51	69.5	76.21
		LIWC	77.87	77.98	77.92	77.92
		Word Uni gram	86.98	83.62	85.27	84.34
		ELMO	88.93	84.68	86.75	84.73
		FastText	90.58	87.87	89.2	89.14
		Latent Semantic Analysis	91.94	89.23	90.56	90.18
		Word2vec	93.54	90.36	91.92	91.1
		BERT-320 (fine-tuned)	93.95	91.22	92.56	92.17
	Char Tri Gram	94.52	92.36	93.43	93.45
With SMOTE ENN Rule	Part-of-Speech	74.32	67.28	76.78	79.58
		LIWC	78.88	79.27	79.07	79.47
		Word Uni gram	87.02	85.54	86.27	86.76
		ELMO	89.28	86.39	87.81	86.48
		FastText	90.84	90.12	90.48	91.07
		Latent Semantic Analysis	92.12	91.03	91.57	91.81
		Word2vec	93.62	91.82	92.71	92.72
		BERT-320 (fine-tuned)	94.19	92.46	93.32	93.93
	Char Tri Gram	94.82	93.01	93.91	94.21

**Table 6:** Impact of class balancing on proposed features for identifying only propaganda class on the test part of the dataset.

**Class balancing**	**Features**	**Precision**	**Recall**	**F1-score**	**AUC**
Without	Part-of-Speech	50.34	9.0	33.93	53.32
		LIWC	79.58	21.63	41.88	60.45
		Word Uni gram	86.95	29.87	45.51	64.63
		ELMO	88.94	29.94	49.59	65.06
		FastText	91.32	30.08	49.48	65.29
		Latent Semantic Analysis	91.75	30.97	51.46	65.46
		Word2vec	93.26	34.36	52.94	66.98
		BERT-320 (fine-tuned)	93.48	44.68	59.73	75.82
	Char Tri Gram	94.45	72.68	81.78	84.39
With Random Oversampling	Part-of-Speech	70.78	64.73	67.62	72.72
		LIWC	77.51	77.83	77.67	77.91
		Word Uni gram	87.03	81.72	84.29	81.93
		ELMO	88.96	82.87	85.81	82.97
		FastText	90.57	85.95	88.2	87.05
		Latent Semantic Analysis	91.98	86.85	89.34	87.89
		Word2vec	93.36	87.32	90.24	88.81
		BERT-320 (fine-tuned)	93.92	87.86	90.79	89.58
	Char Tri Gram	94.47	88.07	91.16	90.67
With SMOTE	Part-of-Speech	72.67	66.23	69.3	76.38
		LIWC	78.19	78.32	78.25	78.12
		Word Uni gram	87.38	83.45	85.37	84.59
		ELMO	89.14	84.38	86.69	84.48
		FastText	90.78	86.37	88.52	89.45
		Latent Semantic Analysis	92.28	88.73	90.47	89.87
		Word2vec	93.63	90.38	91.98	91.18
		BERT-320 (fine-tuned)	94.21	91.21	92.69	92.61
	Char Tri Gram	94.79	91.89	93.32	93.17
With SMOTE ENN Rule	Part-of-Speech	74.58	68.01	77.89	79.96
		LIWC	79.64	79.69	79.87	79.87
		Word Uni gram	87.59	85.78	86.68	87.19
		ELMO	89.78	86.76	88.24	86.89
		FastText	91.46	90.68	91.07	91.83
		Latent Semantic Analysis	92.76	91.64	92.2	92.65
		Word2vec	93.98	92.08	93.02	93.2
		BERT-320 (fine-tuned)	94.4	92.69	93.54	94.37
	Char Tri Gram	95.17	93.69	94.42	94.4

Alternatively, part-of-speech features exhibit the worst performance. It is also important to note that the latest feature methods (ELMO, FastText, LSA, word2vec) performed better than word unigrams, LIWC, and part-of-speech methods. After that, three oversampling methods are applied on the dev and test parts of the dataset, and results are presented in the lower parts of Tables 5 and 6. It is evident that all types of features demonstrated higher values of metrics with SMOTE ENN oversampling as compared to SMOTE and random oversampling methods. We obtained a significant percentage of improvement in all metrics with SMOTE ENN method. In contrast, random oversampling presented the worst performance in comparison with the other two methods. The performance of the char tri-gram and BERT-320 model is comparable at some points. This completes the first objective of the second experiment.

While considering the second objective, the impact of the hybrid combination of the proposed features on identifying propaganda class is reported and analyzed, and compared with two state-of-the-art baselines (baseline 1 (Barrón-Cedeño et al., 2019) and baseline 2 (Kausar, Tahir & Mehmood, 2020)). The investigation is performed on both parts of the dataset (dev and test parts) and results are generated with and without SMOTE ENN oversampling method as shown in Table 7. As SMOTE ENN oversampling outperformed in previous experiments, that’s why only this method is applied here. It is evident from the upper part of Table 7 that all hybrid combinations of proposed features give better evaluation metrics than the two baselines. In addition, we observed improvement in all metrics when hybrid combinations are applied. The combinations of char tri-gram with ELMO, FastText, LSA, Word2vec, and BERT are enlisted. In the precision metric, the best performance is given by the combination of char tri-gram and BERT and achieved the 95.97% on the dev dataset and 96.06% on the test dataset. Moreover, this combination also achieved the highest thresholds for recall, f1-score, and AUC metrics. Here, the pre-trained BERT feature model is used to generate the features, and then these features are combined with the char tri-gram to provide input for the random forest model.

**Table 7:** Comparison of the proposed features (with and without SMOTE ENN oversampling) with the baselines for identifying only propaganda class.

**Over sampling**	**Dataset**	**Features**	**Precision**	**Recall**	**F1-score**	**AUC**
Without	Dev	Baseline 1 [3]	94.7	71.8	81.8	84.9
				Baseline 2 [7]	95.10	71.87	82.06	85.10
				Char Tri Gram + ELMO	95.3	72.3	82.3	85.70
				Char Tri Gram + FastText	95.4	72.4	82.45	85.90
				Char Tri Gram + LSA	95.6	72.6	82.61	86.10
				Char Tri Gram + Word2vec	95.70	73.70	83.30	86.60
			Char Tri Gram + BERT	95.97	74.20	83.89	86.98
		Test	Baseline 1 [3]	94.94	71.92	81.97	85.05
				Baseline 2 [7]	95.31	72.02	82.23	85.38
				Char Tri Gram + ELMO	95.54	72.45	82.47	85.87
				Char Tri Gram + FastText	95.60	72.59	82.58	86.10
				Char Tri Gram + LSA	95.82	72.83	82.74	86.29
				Char Tri Gram + Word2vec	95.87	73.87	83.57	86.89
	Char Tri Gram + BERT		96.06	74.34	84.12	87.13
With SMOTE ENN Over sampling	Dev	Baseline 1 [3]	94.7	71.8	81.8	84.9
				Baseline 2 [7]	95.10	71.87	82.06	85.10
				Char Tri Gram + ELMO	96.45	93.53	94.10	94.42
				Char Tri Gram + FastText	96.67	93.71	94.23	94.54
				Char Tri Gram + LSA	96.79	93.86	94.37	94.67
				Char Tri Gram + Word2vec	96.87	94.08	94.56	94.72
			Char Tri Gram + BERT	96.90	94.17	94.78	94.85
		Test	Baseline 1 [3]	94.94	71.92	81.97	85.05
				Baseline 2 [7]	95.31	72.02	82.23	85.38
				Char Tri Gram + ELMO	96.63	93.71	94.34	94.63
				Char Tri Gram + FastText	96.75	93.82	94.47	94.74
				Char Tri Gram + LSA	96.82	93.94	94.54	94.81
				Char Tri Gram + Word2vec	96.91	94.12	94.68	94.93
	Char Tri Gram + BERT		96.98	94.28	94.79	95.09

We observed an improvement of 1.27% in precision, 2.42% in the recall, 2.15% in the f1-score, and 2.08% in AUC on the dev dataset in comparison with baseline 1. Likewise, improvement of 0.75% in precision, 2.32% in the recall, 1.89% in the f1-score, and 1.75% in the AUC metrics are observed against baseline 2. Thus, the results reported using char tri-gram and BERT are consistent on all evaluation metrics. The second-best performance is observed for both the dev and test datasets when the char tri-gram and word2vec models are combined. This validates the effectiveness of the proposed framework and its outperformance on two baselines. The outperformance of the proposed framework is also justified by all combinations of two-set of features against the baselines. Likewise, the impact of SMOTE ENN oversampling is also validated here and results are shown in the lower part of Table 7 for both dev and test datasets. It is obvious from Table 7 that significant improvement is observed in all combinations of proposed features when SMOTE ENN oversampling is employed. Again char tri-gram with BERT combination outperformed for both dev and test datasets. We observed improvements of 2.04%, 22.36%, 12.82%, and 10.04% in precision, recall, f1-score, and AUC measures against baseline 1 on the test dataset. Similarly, improvements of 1.67%, 22.26%, 12.56%, and 9.71% in precision, recall, f1-score, and AUC are observed against baseline 2 on the test dataset. Almost similar improvement is observed on the dev part of the dataset. This completes the comparison of proposed features with baselines in the presence/absence of oversampling but only for propaganda class.

Moreover, we are interested to examine the influence of proposed features for propaganda and non-propaganda identification as a binary classification model and comparison with two baselines with and without oversampling class balancing. The experimental setup is the same as described earlier. First, we look into the investigation of the impact of individual feature model and their combinations on binary class labels and their comparison with two baselines. It is evident from Table 8 that the char tri-gram has presented the best performance as a standalone model as compared to the other eight feature models. The outperformance of char tri-gram features is also observed at the time of only propaganda class (Tables 5 and 6). The 95.30% precision, 85.70% recall, 89.80% f1-score, and 85.70% AUC achieved by char tri-gram as a standalone model on the dev part is very promising. Likewise, on the test dataset, precision is 95.53%, recall is 85.91%, f1-score is 89.97%, and AUC is 85.87%. The second best-performing feature type is the BERT-320, which also attained this position in the last experiments. Similarly, the third one is the word2vec (same as in the last experiment). In addition, it is now established that the char tri-gram is the most effective type of feature for binary classification as well as for propaganda class.

**Table 8:** Performance comparison (micro-average) of proposed features and baselines.

**Dataset**	**Features**	**Precision**	**Recall**	**F1-Score**	**AUC**
**Dev**	Part-of-Speech	73.70	65.19	70.10	65.0
		LIWC	83.10	69.50	75.80	70.40
		Word Uni gram	89.40	73.40	79.40	74.40
		ELMO	91.10	79.12	83.45	80.34
		FastText	92.40	80.40	85.40	82.40
		Latent Semantic Analysis	92.60	82.90	88.90	84.60
		Word2vec	93.45	83.18	87.67	84.72
		BERT-320	94.67	84.28	88.64	84.97
	Char Tri Gram	95.30	85.70	89.80	85.70
**Test**	Part-of-Speech	73.90	65.35	70.35	65.34
		LIWC	83.31	69.67	75.96	70.68
		Word Uni gram	89.64	73.64	79.68	74.70
		ELMO	91.32	79.34	83.64	80.57
		FastText	92.67	80.68	85.68	82.68
		Latent Semantic Analysis	92.85	83.07	89.07	84.85
		Word2vec	93.72	83.32	87.83	84.91
		BERT-320	94.85	84.46	88.82	85.18
	Char Tri Gram	95.53	85.91	89.97	85.87
**Dev**	Baseline [3]	94.70	81.80	85.60	84.90
		Baseline 2 [7]	94.90	81.97	85.96	85.10
		Char Tri Gram + ELMO	96.60	86.39	90.17	86.07
		Char Tri Gram + FastText	96.90	86.78	90.89	86.78
		Char Tri Gram + LSA	97.10	87.10	91.30	87.10
		Char Tri Gram + Word2vec	97.20	87.70	91.70	87.60
	Char Tri Gram + BERT	97.43	87.93	91.96	87.82
**Test**	Baseline [3]	94.86	81.97	85.83	85.05
		Baseline 2 [7]	95.08	82.08	86.12	85.31
		Char Tri Gram + ELMO	96.82	86.56	90.36	86.26
		Char Tri Gram + FastText	97.08	86.93	91.08	86.93
		Char Tri Gram + LSA	97.26	87.31	91.48	87.27
	Char Tri Gram + Word2vec	97.37	87.87	91.91	87.83
	Char Tri Gram + BERT	97.64	88.10	92.13	88.04

Moreover, we obtained better performance with various combinations of the features in comparison with the two baselines while addressing binary classification. The results are demonstrated in the lower part of Table 8 on the dev and test datasets. It is clearly shown that all combinations of proposed features give better performance than the two baselines. The results are consistent along all evaluation metrics. The char tri-gram + BERT demonstrated the best results on the dev and test parts of the datasets. Likewise, this combination (char tri-gram + BERT) also outperformed when only propaganda class was considered (Tables 5 and 6). In addition, the combination of char tri-gram + word2vec delivered a slightly lower performance than char tri-gram + BERT. We can say that both combinations have comparable performance. The third-best performance is achieved by char tri-gram + LSA.

In the last, we applied SMOTE ENN oversampling method to improve further the performance of the binary classification task and the results are demonstrated in Table 9. It is observed that the performance of various combinations of proposed features delivers higher values in the presence of SMOTE ENN oversampling. But char tri-gram + word2vec presented the best performance here in contrast to char tri-gram + BERT. On the test dataset, 3.44%, 14.81%, 11.7%, and 11.91% improvement in precision, recall, f1-score, and AUC as compared to baseline 1 and 3.22%, 14.7%, 11.41%, 11.65% improvement in precision, recall, f1-score, and AUC in comparison with baseline 2. For binary classification, these thresholds are very promising. Thus, these experiments (Tables 7–9) validate the significance of our proposed framework and demonstrated the outperformance on the two baselines.

**Table 9:** Performance comparison (micro-average) of proposed features and baselines with and without SMOTE ENN oversampling method.

**Class balancing**	**Dataset**	**Features**	**Precision**	**Recall**	**F1-score**	**AUC**
Without	**Dev**	Baseline [3]	94.70	81.80	85.60	84.90
				Baseline 2 [7]	94.90	81.97	85.96	85.10
				Char Tri Gram + ELMO	96.60	86.39	90.17	86.07
				Char Tri Gram + FastText	96.90	86.78	90.89	86.78
				Char Tri Gram + LSA	97.10	87.10	91.30	87.10
				Char Tri Gram + Word2vec	97.20	87.70	91.70	87.60
			Char Tri Gram + BERT	97.43	87.93	91.96	87.82
		**Test**	Baseline [3]	94.86	81.97	85.83	85.05
				Baseline 2 [7]	95.08	82.08	86.12	85.31
				Char Tri Gram + ELMO	96.82	86.56	90.36	86.26
				Char Tri Gram + FastText	97.08	86.93	91.08	86.93
				Char Tri Gram + LSA	97.26	87.31	91.48	87.27
				Char Tri Gram + Word2vec	97.37	87.87	91.91	87.83
	Char Tri Gram + BERT		97.64	88.10	92.13	88.04
With SMOTE ENN OverSampling	**Dev**	Baseline [3]	94.70	81.80	85.60	84.90
				Baseline 2 [7]	94.90	81.97	85.96	85.10
				Char Tri Gram + ELMO	97.76	95.12	96.42	96.23
				Char Tri Gram + FastText	97.84	95.28	96.54	96.38
				Char Tri Gram + LSA	97.96	96.39	97.17	96.54
				Char Tri Gram + BERT	98.10	96.51	97.30	96.78
			Char Tri Gram + Word2vec	98.22	96.62	97.41	96.87
		**Test**	Baseline [3]	94.86	81.97	85.83	85.05
				Baseline 2 [7]	95.08	82.08	86.12	85.31
				Char Tri Gram + ELMO	97.88	95.36	96.6	96.37
				Char Tri Gram + FastText	97.97	95.47	96.7	96.48
				Char Tri Gram + LSA	98.11	96.48	97.29	96.67
				Char Tri Gram + BERT	98.24	96.63	97.43	96.85
	Char Tri Gram + Word2vec		98.30	96.78	97.53	96.96

### Experiment 3: Impact of feature selection on classification performance

One more set of experiments is done to investigate the impact of feature selection for binary classification in the presence of SMOTE ENN rule method. We used two methods for feature selection: (1) the Filter method and (2) the Wrapper method. For the filter method, we used an information gain measure and selected the top 20 features from each category of features. Regarding the wrapper method, we used the forward selection technique with the random forest method (because random forest outperformed in prior experiments). After applying both models, we compare the performance of nine types of features before and after feature selection as shown in Table 10. For comparison, recall, f1-score, and AUC metrics are used.

**Table 10:** Impact of feature selection on the performance of the proposed features and their combinations in the presence of SMOTE ENN oversampling method.

**Data**	**Features**	**Before Selection**			**Filter Method**			**Wrapper Method**		
		**Recall**	**F1**	**AUC**	**Recall**	**F1**	**AUC**	**Recall**	**F1**	**AUC**
**Dev**	Part-of-Speech	77.14	79.94	77.76	76.47	79.52	77.44	78.87	81.06	78.61
		LIWC	79.86	79.81	79.91	78.55	79.51	79.61	80.08	80.12	80.78
		Word Uni-gram	85.87	86.85	86.71	85.94	86.97	86.86	86.02	87.98	86.92
		ELMO	86.87	88.21	86.89	86.89	88.15	86.92	87.01	88.48	87.04
		FastText	90.89	90.93	91.78	90.92	90.96	91.83	91.83	91.10	92.03
		Latent Semantic Analysis	91.78	91.95	92.69	91.80	91.97	92.71	91.97	92.10	92.94
		Word2vec	93.34	93.27	93.31	93.39	93.34	93.41	93.61	93.91	93.78
		BERT-320	94.07	94.47	94.10	94.15	94.52	94.17	94.87	94.72	94.32
	Char Tri Gram	94.78	94.75	94.47	94.83	94.79	94.52	95.05	94.93	94.87
**Test**	Part-of-Speech	77.31	80.12	77.92	77.87	80.41	77.98	79.04	81.21	78.89
		LIWC	79.97	79.95	80.07	80.08	80.06	80.13	80.37	80.41	80.38
		Word Uni-gram	85.98	86.96	86.84	86.02	87.01	86.92	86.28	87.19	87.04
		ELMO	86.97	88.35	86.97	87.00	88.40	87.03	87.12	88.86	87.21
		FastText	91.02	91.09	91.90	91.04	91.14	91.94	92.23	91.35	92.25
		Latent Semantic Analysis	91.93	92.09	92.81	91.97	92.15	92.89	92.27	92.32	93.07
		Word2vec	93.52	93.42	93.48	93.57	93.49	93.52	93.84	93.75	93.79
		BERT-320	94.12	94.61	94.25	94.18	94.67	94.30	94.54	94.87	94.49
	Char Tri Gram	94.86	94.89	94.58	94.92	94.92	94.63	95.17	95.29	94.76
**Dev**	Char Tri Gram + ELMO	95.12	96.42	96.23	87.59	88.74	90.34	97.10	97.34	97.38
		Char Tri Gram + FastText	95.28	96.54	96.38	87.68	88.86	90.48	97.26	97.47	97.51
		Char Tri Gram + LSA	96.39	97.17	96.54	87.79	88.95	90.59	97.38	97.53	97.62
		Char Tri Gram + BERT	96.51	97.30	96.78	87.84	89.10	90.68	97.53	97.64	97.74
	Char Tri Gram + Word2vec	96.62	97.41	96.87	87.92	89.23	90.76	97.64	97.76	97.82
**Test**	Char Tri Gram + ELMO	95.36	96.6	96.37	87.67	88.86	90.51	97.34	97.57	97.47
		Char Tri Gram + FastText	95.47	96.7	96.48	87.78	88.95	90.63	97.42	97.69	97.68
		Char Tri Gram + LSA	96.48	97.29	96.67	87.85	89.07	90.76	97.56	97.75	97.77
		Char Tri Gram + BERT	96.63	97.43	96.85	87.94	89.17	90.84	97.67	97.84	97.86
	Char Tri Gram + Word2vec	96.78	97.53	96.96	88.07	89.28	90.91	97.78	97.93	97.93

The results are reported as follows: First, the impact of nine types of features and their combinations without feature selection are reported. Second, filter method-based feature selection impacts are reported against each type of features and their combinations. Third, the impact of the wrapper-based feature selection method is reported in the last three columns of Table 10. Considering the recall metric, we can observe that the wrapper method demonstrates very higher values for all types of features on the dev and test parts of the dataset. As a standalone model, the char tri-gram attained very higher evaluation metrics using the wrapper method in contrast to the filter method which did not perform well. We obtain significant improvement in all feature types across three evaluation metrics by the wrapper method as compared to the filter method on dev and test datasets. The reason why the char tri-gram presented best is due to the selection of significant 3-chars words that are more helpful in detecting the propaganda content/context in new articles such as bad, met, all, run, etc. In contrast, the filter method failed to identify these dominating features. As BERT is performing the second best, contextual embeddings (for words) selected by wrapper methods are those that are most often found in propaganda articles. For word2vec, only those words are selected by the wrapper method whose vectors are more similar to the words often used in propaganda content. Regarding the latent semantic analysis feature model, the wrapper method selected those 20 topics/themes which are more frequently used in propaganda articles. Similar is the case with FastText and other feature models. Thus, the wrapper method selected an influential set of features and the results (Table 10) demonstrated the effectiveness of the results.

Results on the various combinations of features are demonstrated in the lower part of Table 10 on both the dev and test parts of the dataset. It is evident from the results that the wrapper method demonstrated robust performance as compared to the filter method. All three evaluation metrics show significant improvement in performance for all the combinations. The best performance is obtained by char tri-gram + word2vec with 97.64% recall, 97.76% f1-score, and 97.82% AUC on the dev dataset and with 97.78% recall, 97.93% f1-score, and 97.93% AUC on the test dataset. More than 97% performance against the three evaluation metrics is very promising. In addition, three combinations (char tri-gram + word2vec, char tri-gram + BERT, and char tri-gram + LSA) presented more than 97.50% performance by the wrapper method. But the filter method again did not perform well. The performance improvement demonstrates and proves the effectiveness of the wrapper method as compared to the filter method.

## Discussions and Implications

In the last two decades, the usage and popularity of social media are continuously increasing but unfortunately, these platforms are often exploited by groups or individuals for their vested interests. The propaganda causes serious threats to online users and creates panic and distrust. The findings of this study help us to uncover the significant type of features and their combinations to effectively identify propaganda in news articles. The current study has valuable insights for news channel owners, online readers, and law-and-enforcement organizations to early detect propagandistic content from news articles. In today’s world, online tools for detecting propaganda content in news articles and social media are in high demand. So the development of such types of tools is mandatory both for the mass media community and lay readers. In this regard, we have designed a detection system for propaganda by exploiting more significant content, language-based, and word embeddings-based feature models with a random forest ML model. The proposed system was evaluated on a real-life news articles dataset so that a more practical system could be developed. The most significant contribution is the embodiment of semantic and language-based feature vectors in the identification system that achieves 97.64% recall and improves 15.70% recall as compared to two standard baselines.

Our research has few practical implications. The findings of this study can be utilized to design a filter for online social media forums so that propagandistic content can be identified early and the system may discard unwanted content. It has been observed that propagandistic contents have a strong impact on politics and religious events. Almost all political parties use various types of propaganda against their opponents to beat them in election campaigns and on other occasions. In the same way, religious followers also take advantage of propaganda in conflicting opinions. Although our framework did not support the identification of the target of propaganda campaigns and the source of propaganda spreading groups or individuals. Therefore, target identification and source identification of propagandistic contents must be done. In addition, our proposed framework can be utilized in other related domains to develop an effective system.

Moreover, there are few limitations of this research. We used the Qprop dataset (Barrón-Cedeño et al., 2019) for experimentation in which the train, dev, and test parts are there. To our point of view, the size of the dataset is not large enough to generalize the findings on other news platforms’ articles. In the future, we may design a new dataset that should be large enough. Future work may incorporate this limitation to improve performance.

## Conclusion and Future Work

This study performed a comprehensive set of experiments to detect propaganda in news articles. The proposed framework is validated on a publicly available Qprop dataset. Among the applied ML methods, the random forest ML model outperformed and demonstrated very promising results. Three oversampling techniques are explored to handle the imbalanced state of the dataset. The fine-tuning of BERT is also done and we obtained a BERT-320 model, that demonstrated 94.88%, and 95.10% precision on the dev and test datasets respectively for binary classification in the presence of SMOTE ENN oversampling method. As a standalone model, the char tri-gram has demonstrated the best performance (achieved 93.69% recall for the propaganda class and 94.78% for the binary classification). As a hybrid combination, char tri-gram and BERT-320 are more effective than any other combinations of proposed features for the propaganda class identification task. Moreover, for binary classification in the presence of SMOTE ENN oversampling, char tri-gram + wor2vec presented the best performance and achieved 96.78% recall. Different from prior approaches, we used the filter and wrapper methods for the selection of the best subset. The wrapper method demonstrated better performance than the filter method by selecting a significant subset of features from each category of features. We achieved more than 97.50% performance in the recall, f1-score, and AUC respectively with the wrapper method. Thus, our framework in the presence of class balancing and feature selection is more robust to detect propaganda from news articles.

This research can be extended in multiple directions such as utilizing more effective and domain-specific features for propaganda identification. Another venue for research is to develop a rule-based framework using Sentic computing. The latest language models can also be utilized to enhance the performance of the current framework.

##  Supplemental Information

10.7717/peerj-cs.1248/supp-1 File S1 Python Code

10.7717/peerj-cs.1248/supp-2 Supplemental Information 2 LIWC features

10.7717/peerj-cs.1248/supp-3 Supplemental Information 3 Word2vec features

10.7717/peerj-cs.1248/supp-4 Supplemental Information 4 POS Features

10.7717/peerj-cs.1248/supp-5 Supplemental Information 5 LSA Features

10.7717/peerj-cs.1248/supp-6 Supplemental Information 6 FastText features

10.7717/peerj-cs.1248/supp-7 Table S1 Summary of prior work to detect propaganda in the text

## Additional Information and Declarations

### Data Availability

The following information was supplied regarding data availability: The data and code are available in the Supplemental Files.
