# Supplementary material for: How to detect propaganda from social media? Exploitation of semantic and fine-tuned language models
Source: PeerJ Comput Sci. 2023 Feb 20;9:e1248. doi: 10.7717/peerj-cs.1248 (PMC10280574; doi:10.7717/peerj-cs.1248)
Supplement: Table S1 [file peerj-cs-09-1248-s007.docx]

| Platform | Language | Features | Classifier |
| --- | --- | --- | --- |
| News Articles (Horne & Adali 2017) | English | Stylistic, complexity, and Psychological Features | Linear kernel SVMs |
| Wikipedia (Thorne et al. 2018) | English | TF-IDF | MLP |
| CQA forums (Mihaylova et al. 2019) | English | Linguistic Stylistic | BERT , LSTM |
| News Media (Rashkin et al. 2017) | English | LIWC | LSTM, Naive Bayes |
| Twitter (Shao et al. 2017) | English | User-based, network, temporal and Sentiment | RF |
| Twitter (Tundis et al. 2021) | Mixed-code | Single, multiple, and third-party scripts, Special Characters for Alphabets | Multinomial Naive Bayes, SVM, LR, CNN |
| News Articles (Hardalov et al. 2016) | English | linguistic, sentiment, semantic features | LR |
| News Articles (Potthast et al. 2017) | English | POS, char n-gram, lexicon features, readability | RF |
| News Articles (Baly et al. 2018) | English | Structure, Sentiment, Complexity, and Morality | SVM |
| Social Network (Canini et al. 2011) | English | TF-IDF | LDA |
| Twitter (Ba et al. 2016) | English | Entity relation | Estimation-based |
| Social Media (Chen et al. 2013) | Chinese | Semantic, metadata features | SVM |
| News Articles (Kulkarni et al. 2018) | English | multi-modal and network features | LR, CNN, FNN |
| Tweets (Guo & Vosoughi 2021) | English | Organizational and topic features | BERT, CNN |
| News (Khanday et al. 2021) | English | TF/IDF, BOW | SVM |
| News Articles (Li et al. 2021) | English | Sentence level features | BERT |
| Tweets (Han et al. 2020) | English | User features (Followers, friends, Lists etc) | GNN |
| Tweets (Nouh et al. 2019) | English | Psychological, behavioral and textual features | RF |
| News Articles (Da San Martino et al. 2019) | English | Fragment and sentence level features | Neural network |
| News Articles (Horne et al. 2018) | English | Content-based features, TF-IDF | Linear kernel SVM |
| English Books (Koppel et al. 2007) | English | linguistiuc, Syntactic, complexity and richness | Linear kernel SVM |
| News Articles (Popat et al. 2017) | English | Linguistic Features, unigrams and bigrams | L2 regularized LR |
| News (Mihaylov et al. 2015) | Bulgarian, English | Vote, comment and time based features | SVM |
| News Articles (Zuo et al. 2018) | English | Lexical, stylometric, semantic and affective | MLP,SVM |
| News Articles (Hansen et al. 2018) | English | Sentiment, TF-IDF, POS, Entity extraction | RNN Model |
| News (Huang et al. 2022) | English | BERT and ROBERTA features | MLP, HDSF |
| New Articles (Polonijo et al. 2021) | English | Word2vec, sentiment and hybrid features | Deep Learning |
| New Articles (Yu et al. 2021) | English | Syntactic, sentiment, structural | Multi-granularity Net |
| News (Bagdasaryan & Shmatikov 2021) | English | Sentiment, toxicity, entailment, word Embedding | RoBERTa |
| News (Barfar 2022) | English | linguistic features | Light GBM |
| News Articles (Oliinyk et al. 2020) | English | Tf-IDF, POS, Lexicons based and Word2vec | LR |
| News Articles (Altiti et al. 2020) | English | Word2vec word embedding | BERT |
| News Articles (Barrón-Cedeño et al. 2019) | English | NELA, word and char n-gram, LIWC features | Max-Entropy classifier |
